# Numerical Investigation of Dynamic Wrinkling Behaviors in Stiff-Film/PDMS-Substrate Structure

**DOI:** 10.3390/polym18020292

**Published:** 2026-01-21

**Authors:** Haohao Bi, Wenjie Li, Liuyun Wang, Bo Wang

**Affiliations:** 1 School of Science, Qingdao University of Technology, Qingdao 266520, China; 2 Department of Engineering Mechanics, Northwestern Polytechnical University, Xi’an 710072, China

**Keywords:** dynamic behaviors, polydimethylsiloxane substrate, checkerboard wrinkling mode

## Abstract

Thin film/substrate structures based on the principle of buckling mechanics exhibit both excellent stretchability and mechanical stability, and they have been recognized as a critical configuration in the design of flexible electronic devices. During application, flexible electronic devices are usually subjected to complex dynamic environments. Therefore, it is of great significance to investigate the dynamic behavior of thin film/substrate structures for the design of flexible electronic devices. The bending energy, membrane energy, and kinetic energy of the thin film and the elastic energy of the substrate were calculated. On this basis, the dynamic equation of the thin film/substrate structure with a checkerboard wrinkled pattern was derived by applying the principle of minimum energy combined with the Lagrangian function. Numerical simulations were conducted on the system to analyze the effect of pre-strain and the Young’s modulus of substrate on the system’s potential energy function, simulate the temporal response of the system’s dynamic behavior, and investigate the influences of pre-strain and the Young’s modulus of substrate on system stability and the chaos critical value. Theoretical support is expected to be provided for the design of two-dimensional (2D) thin film/substrate structures through this research.

## 1. Introduction

Based on the principle of buckling mechanics, the wrinkled structure of a thin, stiff film and polydimethylsiloxane(PDMS) substrate possesses both excellent stretchability and mechanical stability. This thin film/substrate structure has become a key configuration in the design of flexible electronic devices, demonstrating broad application potential in materials science and emerging functional device fields. Therefore, it is necessary to study the dynamic response of this structure under dynamic loading, as it not only reveals the instability evolution of this structure but also provides theoretical support for improving the structural reliability and service performance of flexible electronics. This is of great significance for promoting the design and engineering application of such devices [1,2,3].

With respect to the static buckling problem of film/substrate structures, extensive investigations have been carried out by numerous scholars in the field. In the research on the formation and evolution of film wrinkles, remarkable advancements have been achieved in multiple studies. Based on the principle of energy minimization, Huang et al. [4] investigated the wrinkling behavior in a multilayer structure consisting of a rigid film, a soft substrate, and a rigid support. To accurately predict the instability behavior of practical devices under tension, Li et al. [5] developed an analytical model capable of distinguishing between global buckling and local wrinkling for substrates with finite thickness. To reveal the critical influence of substrate nonlinearity on advanced wrinkling modes, Zang et al. [6] analyzed the buckling behavior of rigid films on pre-stretched elastic substrates. A theoretical framework for film buckling on soft substrates has been developed by Pan et al. [7], which clarifies the transition path and geometric laws from a flat state to a blister state under uniaxial and equibiaxial conditions. For the first time, the crystallographic anisotropy of films and the viscoelasticity of substrates have been coupled by Im et al. [8], and the entire process from the initiation of instability to the formation of equilibrium patterns has been elaborated. To investigate the factors influencing the evolution of wrinkles, Jin et al. [9] examined how the relative magnitude between the critical strain for wrinkling and the critical strain for wrinkling initiation affects the formation and evolution of wrinkles.

Further investigations into two-dimensional buckling have been conducted by numerous scholars. In terms of the buckling behavior in the hard film/soft substrate system driven by thermal stress, it has been investigated by Song et al. [10] that three buckling modes, namely one-dimensional, checkerboard, ordered herringbone modes, and a prediction model based on the film and various parameters, have been established. For the biaxial structure of silicon nanomembranes with two-dimensional buckling, Choi et al. [11] have studied the geometric shape and strain response of the material along different directions. By applying orthogonal strain to the interlayer of an elastomer, Pellegrino et al. [12] developed a method for generating two-dimensional dual-frequency patterns through the superposition of one-dimensional single-frequency wrinkles. Based on the buckling behavior of elastic films under residual compression, the interface boundary conditions of film/substrate have been analyzed, and linear stability analysis has been performed by Audoly et al. [13]. To optimize the design of functional surfaces in flexible electronics, Huang et al. [14] identified that the tension–compression asymmetry of soft substrates serves as the key factor governing pattern evolution. To investigate the difference in buckling behavior under uniaxial and biaxial compression, Peterson et al. [15] conducted a quantitative comparison of the buckling behavior in silicon-germanium thin films under these two loading conditions and contrasted their respective buckling propagation rates.

In the research on the nonlinear behavior of film buckling, significant contributions have been made by numerous scholars to the exploration of its analytical or numerical solutions. Regarding the formation mechanism of film wrinkle patterns on planar and three-dimensional structured substrates, a hybrid analytical-numerical method has been proposed by Cutolo et al. [16] to simulate the wrinkle evolution process. Based on three-dimensional nonlinear finite element analysis, Xu et al. [17] explored the pattern formation process of rigid films on flexible substrates, and they adopted the asymptotic numerical method (ANM) to achieve robust path following and bifurcation detection. Based on a neo-Hookean substrate under uniaxial compression, the buckling behavior of neo-Hookean films has been studied by Fu et al. [18], and it has been revealed that there exists a class of local solutions with asymptotically decaying tails in the subcritical region. Under two-dimensional strain conditions, Nikravesh et al. [19] proposed a finite element method to simulate the wrinkle formation in thin films on flexible substrates, where random initial imperfections were introduced to trigger instabilities. Based on the finite deformation theory, the analytical solution for the highly nonlinear behavior of the mechanical model of buckled films on soft substrates has been obtained by Song et al. [20] through perturbation analysis. In the field of flexible electronic engineering, the precise regulation of surface patterns of film/substrate structures serves as a crucial design basis for the realization of device functionalities. To explore the variation in buckling patterns, Audoly et al. [21] investigated the buckling behavior of compressed elastic films and revealed the evolutionary path of buckling patterns during the accumulation of residual stresses. To reveal the energy characteristics of different wrinkles, Cai et al. [22] compared the energetic properties of various periodic wrinkle patterns formed in thin rigid films on biaxially compressed compliant substrates over a wide range of overstress. With respect to the influence of surface patterns on the energy of film/substrate structures, the effect of equibiaxial compressive stress on modes has been studied by Chen et al. [23], and it has been revealed that the herringbone mode is the configuration with the lowest energy. Based on the multi-scale method with slowly varying Fourier coefficients, Xu et al. [24] investigated the pattern formation of rigid thin films on soft substrates and realized the quantitative description and bifurcation analysis of sinusoidal and checkerboard patterns through a multiscale finite element framework. To predict buckling orientation, Song et al. [25] developed a theoretical model for chevron buckling patterns in anisotropic thin films on elastic substrates.

When film/substrate electronic devices operate in complex environments, it is of great significance to investigate their nonlinear dynamic behaviors to ensure the performance and reliability of the devices. Based on the analytical solution of Jacobi elliptic functions, Ou et al. [26] investigated the dynamic buckling behavior of thin films on soft substrates and determined the dynamic critical load by incorporating the Budiansky–Roth criterion. By employing the symplectic method, the nonlinear dynamic behaviors of piezoelectric film/soft substrate structures, as well as the random vibration behaviors under white noise excitation, have been analyzed by Wang et al. [27] and Bi et al. [28]. Considering the influence of the damping effect on film/substrate structures, a multi-level coupled time-varying parameter dynamic model has been developed by Zhou et al. [29] to analyze the nonlinear dynamic buckling of film/viscoelastic substrate structures under time-varying excitation. By introducing the dynamic boundary effect of sub-nanoscale van der Waals interactions, a dynamic analysis model for layered nanofilms on substrates has been established by Dong et al. [30]. Based on the small deformation theory, the governing equations have been derived by Zhang et al. [31] through the Lagrangian function and Euler-Lagrange equations, and the analytical solution of the response under linear loads has been obtained. To investigate the dynamic characteristics of buckling piezoelectric energy harvesters on pre-strained compliant substrates, Bi et al. [32] established governing equations and solved for steady-state responses, revealing that pre-strain and substrate modulus can effectively modulate high-energy orbits. Based on the nonlinear free vibration of beams on Winkler foundations, Ma et al. [33] established nonlinear equations of motion accounting for soil-structure dynamic coupling, and they obtained both linear and nonlinear natural frequencies and mode shapes of the beams through eigenvalue analysis and the multiple scales method. Based on the buckling behavior of thin films under nanoscale surface effects, Wang et al. [34] analyzed the influence of surface elasticity and residual surface tension on static and dynamic characteristics, and proposed design strategies for buckled film interconnects in flexible electronics. For the three-layer structure, Wang et al. [35] established static and dynamic theoretical models for the film/interlayer/substrate system, and the buckling amplitude and nonlinear vibration frequency have been analyzed. To elucidate the role of interlayers in regulating wrinkle stability, Bi et al. [36] investigated their influence on the system’s buckling behavior, distinguishing between global buckling and local wrinkling modes.

It has been widely documented that in film/substrate structure, two-dimensional buckling modes, including herringbone and checkerboard patterns, have been extensively investigated under static conditions. Nevertheless, it should be emphasized that their mechanical response under dynamic loading remains inadequately explored. Consequently, the checkerboard buckling mode is selected as the research focus in this study, and its vibration characteristics are systematically examined. In Section 2 of the paper, the dynamic equations of the thin film/substrate structure with a checkerboard wrinkle pattern are derived. In Section 3, numerical simulations are performed on the dynamic system, and the effects of parameters such as pre-strain on the dynamic behaviors of the system are discussed. Finally, in Section 4, conclusions are drawn.

## 2. Materials and Methods

**Figure 1:**
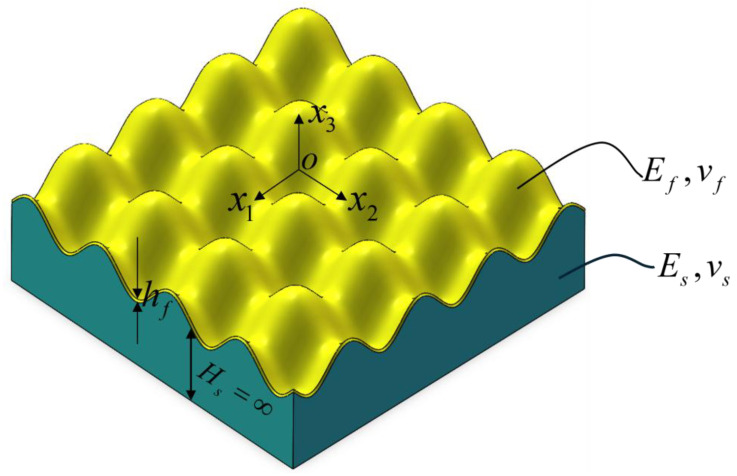
Schematic diagram of film/substrate structure.

As shown in Figure 1, for the checkerboard film/substrate structure, $h_{f}$ denotes the thickness of the film, $v_{f}$ and $v_{s}$ represent the Poisson’s ratios of the film and the substrate, respectively, and $E_{f}$ and $E_{s}$ denote the Young’s moduli of the film and the substrate, respectively. In this paper, it is assumed that ${\bar{E}}_{f}=E_{f}/1-v_{f}^{2}$ and ${\bar{E}}_{s}=E_{s}/1-v_{s}^{2}$ are the equivalent Young’s modulus of the film and the equivalent Young’s modulus of the substrate, respectively. The $x_{1}$ and $x_{2}$ axes point to the principal strain directions of the biaxial load, respectively, and the $x_{3}$ direction represents the thickness direction of the film. The symbol explanations in the text are also uniformly presented in Table A1 of Appendix A. The thickness of the substrate is sufficiently larger than that of the film, and it is regarded as a semi-infinite space in the model.

According to Huang et al. [4] and Song et al. [10], assuming the out-of-plane displacement function is given by

(1) $$w=A\left(t\right)\cos(k_{1}x_{1})\cos(k_{2}x_{2}),$$

where w denotes the out-of-plane displacement of the checkerboard membrane structure, $A\left(t\right)$ represents the time-varying wrinkle amplitude of this structure, and $k_{1}$ and $k_{2}$ are the characteristic wave numbers along the $x_{1}$ and $x_{2}$ directions, respectively. The bending energy density of the film is expressed as Huang et al. [4] and Song et al. [10]

(2) $$W_{b}=\frac{{\bar{E}}_{f}h_{f}^{3}}{24}\left[{\left(\frac{\partial^{2}w}{\partial x_{1}^{2}}\right)}^{2}+{\left(\frac{\partial^{2}w}{\partial x_{2}^{2}}\right)}^{2}+2v_{f}\frac{\partial^{2}w}{\partial x_{1}^{2}}\frac{\partial^{2}w}{\partial x_{2}^{2}}+2(1-v_{f}){\left(\frac{\partial^{2}w}{\partial x_{1}\partial x_{2}}\right)}^{2}\right].$$

By computing the second-order partial derivatives of w with respect to $x_{1}$ and $x_{2}$, along with the mixed partial derivative with respect to $x_{1}x_{2}$, and substituting these into Equation (2), the bending energy density can be expressed in terms of A, $k_{1}$, and $k_{2}$ as

(3) $$W_{b}=\frac{A^{2}\left(t\right){\bar{E}}_{f}h_{f}^{3}}{24}\left[\begin{array}{l} k_{1}^{4}\cos^{2}(k_{1}x_{1})\cos^{2}(k_{2}x_{2})+k_{2}^{4}\cos^{2}(k_{1}x_{1})\cos^{2}(k_{2}x_{2})\\ +2v_{f}{k_{1}}^{2}{k_{2}}^{2}\cos^{2}(k_{1}x_{1})\cos^{2}(k_{2}x_{2})\\ +2(1-v_{f}){k_{1}}^{2}{k_{2}}^{2}\sin^{2}(k_{1}x_{1})\sin^{2}(k_{2}x_{2}) \end{array}\right].$$

Then, the bending energy of the film is

(4) $$U_{b}=\frac{k_{1}}{2\pi }\frac{k_{2}}{2\pi }\int_{0}^{2\pi /k_{1}}\int_{0}^{2\pi /k_{2}}W_{b}dx_{2}dx_{1}.$$

Substituting Equation (3) into Equation (4) yields

(5) $$U_{b}=\frac{{\bar{E}}_{f}h_{f}^{3}}{96}{\left(k_{1}^{2}+k_{2}^{2}\right)}^{2}A^{2}\left(t\right).$$

Since the shear stress at the film/substrate interface has no effect on the wavelength and amplitude of the structure, i.e., the shear stress at the interface is zero, it follows that the shear stress in different directions can be obtained as

(6) $$T_{i}=\frac{\partial N_{i1}}{\partial x_{1}}+\frac{\partial N_{i2}}{\partial x_{2}}=0,$$

where $N_{ij}$ are the membrane forces to the membrane strains, and can be obtained as

(7) $$N_{ij}=h_{f}{\bar{E}}_{f}\left[\left(1-v_{f}\right)\varepsilon_{ij}+v_{f}\left(\varepsilon_{11}+\varepsilon_{22}\right)\delta_{ij}\right],$$

(8) $$\varepsilon_{ij}=\varepsilon_{ij}^{pre}+\frac{1}{2}\left(\frac{\partial u_{i}}{\partial x_{j}}+\frac{\partial u_{j}}{\partial x_{i}}\right)+\frac{1}{2}\frac{\partial w}{\partial x_{i}}\frac{\partial w}{\partial x_{j}}.$$

where $\delta_{ij}=\left\{\begin{array}{l} 1,i=j\\ 0,i\neq j \end{array}\right.$, $\varepsilon_{ij}$ denotes the strain tensor of the film, i,j=1,2, and $\varepsilon_{ij}^{pre}$ represents the pre-strain of the thin film in each direction.

The relationships between the in-plane displacement of the film $u_{1}^{f}$, $u_{2}^{f}$ and $x_{1}$, $x_{2}$ can be obtained, leading finally to

(9) $$u_{1}^{f}(x_{1},x_{2})=\frac{A^{2}\left(t\right)\left[2k_{1}^{2}\cos^{2}(k_{2}x_{2})-v_{f}k_{2}^{2}\right]}{16k_{1}}\sin(2k_{1}x_{1}),$$

(10) $$u_{2}^{f}(x_{1},x_{2})=\frac{A^{2}\left(t\right)\left[2k_{2}^{2}\cos^{2}\left(k_{1}x_{1}\right)-v_{f}k_{1}^{2}\right]}{16k_{2}}\sin(2k_{2}x_{2}).$$

The membrane energy density of the film is

(11) $$W_{m}=\frac{1}{2}(N_{11}\varepsilon_{11}+N_{12}\varepsilon_{12}+N_{21}\varepsilon_{21}+N_{22}\varepsilon_{22}).$$

Substituting Equations (1), (9) and (10) into Equation (8), $W_{m}$ can then be determined.

(12) $$U_{m}=\frac{k_{1}}{2\pi }\frac{k_{2}}{2\pi }\int_{0}^{2\pi /k_{1}}\int_{0}^{2\pi /k_{2}}W_{m}dx_{2}dx_{1},$$

The membrane energy can then be calculated as

(13) $$U_{m}=\frac{{\bar{E}}_{f}h_{f}}{256}\left[\begin{array}{l} A^{4}(3-v_{f}^{2})(k_{1}^{4}+k_{2}^{4})+4v_{f}A^{4}k_{1}^{2}k_{2}^{2}-32A^{2}(k_{1}^{2}+v_{f}k_{2}^{2})\varepsilon_{11}^{pre}\\ -32A^{2}(k_{2}^{2}+v_{f}k_{1}^{2})\varepsilon_{22}^{pre}+128({\varepsilon_{11}^{pre})}^{2}+128({\varepsilon_{22}^{pre})}^{2}+256v_{f}\varepsilon_{11}^{pre}\varepsilon_{22}^{pre} \end{array}\right].$$

The strain energy density of the substrate is

(14) $$W_{s}=\frac{1}{2}(\sigma_{11}\varepsilon_{11}+\sigma_{22}\varepsilon_{22}+\sigma_{33}\varepsilon_{33}),$$

where $\sigma_{ij}$ denotes the stress of the substrate.

And the strain energy of the substrate is obtained as

(15) $$\begin{array}{c} U_{s}=\frac{k_{1}}{2\pi }\frac{k_{2}}{2\pi }\int_{0}^{2\pi /k_{1}}\int_{0}^{2\pi /k_{2}}\int_{0}^{\infty }W_{s}dx_{3}dx_{2}dx_{1}\\ =\frac{{\bar{E}}_{s}}{16}\sqrt{k_{1}^{2}+k_{2}^{2}}A^{2}\left(t\right)\;\;\;\;\;\;\; \end{array}.$$

This study employs the principle of energy minimization. By taking the partial derivatives of the total energy $U_{total}=U_{b}+U_{m}+U_{s}$ with respect to A, $k_{1}$, $k_{2}$, and setting them to zero, the wave numbers ($k_{1}=k_{2}$) are finally obtained as [10]

(16) $$k_{1}=k_{2}=\frac{1}{\sqrt{2}}\frac{1}{h_{f}}{\left(\frac{3{\bar{E}}_{s}}{{\bar{E}}_{f}}\right)}^{1/3}.$$

The total kinetic energy of the film/substrate structure is

(17) $$\begin{array}{c} E=\frac{1}{2}\rho_{f}h_{f}\frac{k_{1}}{2\pi }\frac{k_{2}}{2\pi }\int_{0}^{2\pi /k_{1}}\int_{0}^{2\pi /k_{2}}{\left(\frac{\partial w}{\partial t}\right)}^{2}dx_{2}dx_{1}\\ =\frac{1}{8}\rho_{f}h_{f}{\dot{A}}^{2}\left(t\right)\;\;\;\;\;\;\;\;\;\;\; \end{array},$$

where $\rho_{f}$ is the density of the film. The work performed by external forces is

(18) $$U_{work}=\frac{k_{1}}{2\pi }\frac{k_{2}}{2\pi }\int_{0}^{2\pi /k_{1}}\int_{0}^{2\pi /k_{2}}F_{m}\left(x_{1},x_{2},t\right)wdx_{1}dx_{2}$$

where $F_{m}\left(x_{1},x_{2},t\right)=f_{m}\left(t\right)\cos\left(k_{1}x_{1}\right)\cos\left(k_{2}x_{2}\right)$

The Lagrangian function of this structure is $L=E-U_{total}$, and the Euler equation is given by

(19) $$\frac{d}{dt}\left(\frac{\partial L}{\partial \dot{A}}\right)-\frac{\partial L}{\partial A}=\frac{\partial U_{\mathrm{work}}}{\partial A},$$

Taking linear damping into account for this system, the dynamic equation of this structure can be derived as

(20) $$\frac{1}{4}\rho_{f}h_{f}\ddot{A}\left(t\right)+c\dot{A}\left(t\right)+\alpha_{1}A\left(t\right)+\alpha_{2}A^{3}\left(t\right)=\frac{1}{4}f_{m}\left(t\right),$$

where c is the damping term. $f_{m}\left(t\right)$ is the external excitation, $f_{m}\left(t\right)=f\cos\left(2\pi \omega t\right)$, f is the amplitude of the external excitation, ω is the frequency of the external excitation, and

$$\begin{array}{l} \alpha_{1}=\frac{{\bar{E}}_{f}h_{f}^{3}}{48}{\left(k_{1}^{2}+k_{2}^{2}\right)}^{2}+\frac{{\bar{E}}_{s}}{8}\sqrt{k_{1}^{2}+k_{2}^{2}}-\frac{{\bar{E}}_{f}h_{f}}{4}\left[(k_{1}^{2}+v_{f}k_{2}^{2})\varepsilon_{11}^{pre}+(k_{2}^{2}+v_{f}k_{1}^{2})\varepsilon_{22}^{pre}\right],\\ \alpha_{2}=\frac{{\bar{E}}_{f}h_{f}}{64}\left[(3-v_{f}^{2})(k_{1}^{4}+k_{2}^{4})+4v_{f}k_{1}^{2}k_{2}^{2}\right] \end{array}.$$

For convenience, the following nondimensional parameters of this structure are introduced,

$$\bar{A}=\frac{A}{h_{f}},\;\tau =th_{f}\sqrt{\frac{\alpha_{2}}{\frac{1}{4}\rho_{f}h_{f}}},$$

Substitution of the above equations into the system (20) yields

(21) $$\frac{d^{2}\bar{A}}{d\tau^{2}}+\bar{c}\frac{d\bar{A}}{d\tau }+\frac{\alpha_{1}}{{h_{f}}^{2}\alpha_{2}}\bar{A}+{\bar{A}}^{3}={\bar{f}}_{m}\left(\tau \right)$$

where ${\bar{f}}_{m}\left(\tau \right)=\bar{f}\cos\left(2\pi \bar{\omega }\tau \right)$.

The potential energy function of the system (21) is

(22) $$\bar{W}=\frac{\alpha_{1}}{2{h_{f}}^{2}\alpha_{2}}{\bar{A}}^{2}+\frac{{\bar{A}}^{4}}{4}.$$

## 3. Results and Discussion

### 3.1. Comparison with Experiment

**Figure 2:**
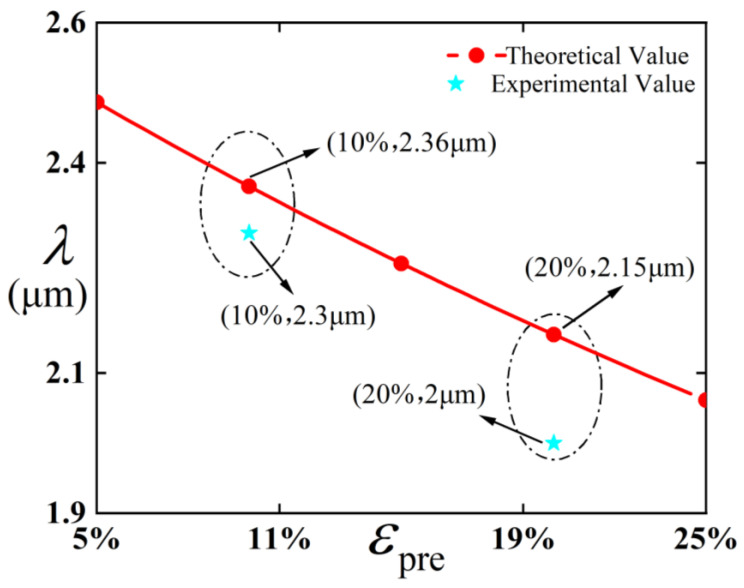
Dependence of the wavelength on pre-strain.

To enable a more specific comparison with previous experiments, this study presents plots of both wavelength and amplitude versus pre-strain at an evolution time of 50 s, which are compared with the experimental results reported in Pellegrino et al. [12]. The comparison is illustrated in Figure 2, and the parameters used in this work are listed as follows: ${\bar{E}}_{f}=2\mathrm{GPa}$, ${\bar{E}}_{s}=2.1\mathrm{MPa}$, $h_{f}=43.2\mathrm{nm}$.

Figure 2 is compared with the amplitude and wavelength at an evolution time of 50 s in Pellegrino et al. [12]. As shown in Figure 2, when the pre-strain is 10%, the theoretical wavelength is 2.36 μm, while the actual value reported in Pellegrino et al. [12] is 2.3 μm, resulting in a relative error of 2.6%. When the pre-strain is 20%, the theoretical wavelength is 2.15 μm, compared to the actual value of 2 μm in Pellegrino et al. [12], yielding a relative error of 7.5%. Both cases show relatively small discrepancies. These results further corroborate the accuracy of the theoretical derivations presented in this paper.

### 3.2. The Effect of the Pre-Strain

**Figure 3:**
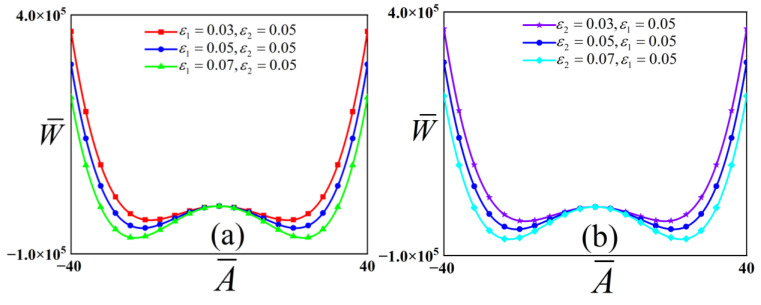
Potential energy functions of the checkerboard buckling mode structure. Panel (**a**) corresponds to a fixed value of $\varepsilon_{2}=0.05$, examining the influence of varying $\varepsilon_{1}$ Panel (**b**) corresponds to $\varepsilon_{1}=0.05$, showing the effect of different $\varepsilon_{2}$ values on the potential.

To illustrate the dynamic energy characteristics of the buckling system, we examine the potential energy function of a structure exhibiting a checkerboard buckling mode. The influence of the pre-strain parameters on this potential energy function is explored, with the results visualized in Figure 3. The other physical parameters of the structure with a checkerboard buckling mode are defined as Song et al. [10] $E_{f}=130\;\mathrm{GPa}$, $v_{f}=0.27$, $E_{s}=1.8\;\mathrm{MPa}$ $v_{s}=0.48$, $h_{f}\;=10^{-7}\;m$.

As shown in Figure 3, the system’s potential energy function possesses a single saddle point and two stable equilibrium points. In Figure 3a, with $\varepsilon_{2}=0.05$, an increase in $\varepsilon_{1}$ causes the positions of the stable equilibria to shift away from the initial dimensionless displacement. Concurrently, the potential barrier height increases monotonically. Furthermore, the distance between the two stable equilibria and the separation distance between the wells would increase with increasing $\varepsilon_{1}$. Similarly, Figure 3b demonstrates that for $\varepsilon_{1}=0.05$, increasing $\varepsilon_{2}$ also shifts the stable equilibria away from the initial displacement, elevates the potential barrier, and increases the separation between the two potential wells. These observations indicate that the pre-strain parameters $\varepsilon_{1}$ and $\varepsilon_{2}$ exert a symmetrical impact on the potential energy landscape. Therefore, we only need to discuss the influence of the value of $\varepsilon_{1}$ on the dynamic behavior.

**Figure 4:**
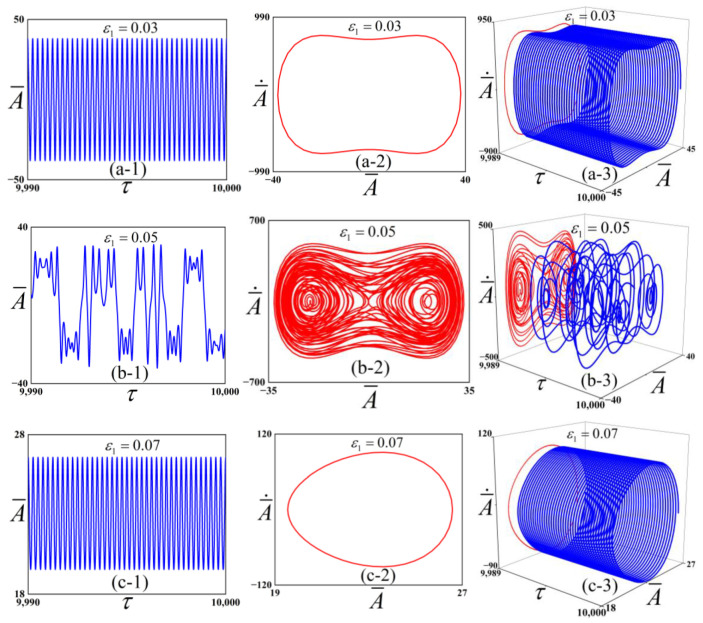
Simulation results of the checkerboard buckling mode structure: (**a-1**), (**a-2**), and (**a-3**), respectively, show the dimensionless displacement time response, phase portrait, and three-dimensional projection for $\varepsilon_{1}=0.03$; (**b-1**–**b-3**) correspond to the dimensionless displacement time response, phase portrait, and three-dimensional projection for $\varepsilon_{1}=0.05$; (**c-1**–**c-3**) represent the dimensionless displacement time response, phase portrait, and three-dimensional projection for $\varepsilon_{1}=0.07$.

Driven by the aim to preliminarily investigate the influence of the parameter $\varepsilon_{1}$ on the system’s dynamic behavior, we present the numerical results in Figure 4. The parameter $\varepsilon_{1}$ was varied across the values 0.03, 0.05, and 0.07.

Figure 4 shows three different oscillating modes with $\bar{f}=1300$, $\bar{c}=0.01$, $\bar{\omega }=4$. For $\varepsilon_{1}=0.03$, the system exhibits a periodic response with one dominant cycle. At this point, combined with Figure 3, the potential well depth is relatively small, the energy required to cross the potential well is relatively low, and inter-well vibration is prone to occur. When $\varepsilon_{1}$ increases to 0.05, the system transitions into a chaotic state, characterized by an aperiodic and irregular time-history response of the dimensionless displacement. In this case, the system can vibrate in cross-well. Further increasing $\varepsilon_{1}$ to 0.07 restores a periodic oscillation. As $\varepsilon_{1}$ increases, the potential well depth increases, and the energy required to cross the potential well becomes larger; at this point, the system undergoes intra-well vibration.

**Figure 5:**
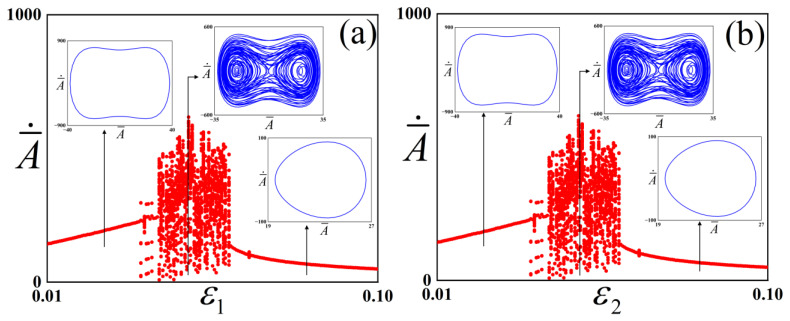
Bifurcation diagrams of the checkerboard buckling mode structure with respect to the pre-strain parameters: (**a**) system response to variations in $\varepsilon_{1},\varepsilon_{2}=0.05$, (**b**) system response to variations in $\varepsilon_{2},\varepsilon_{1}=0.05$. The red dots represent the bifurcation diagrams, while the blue lines represent the phase diagrams under different parameters.

In order to investigate the influence of the pre-strain parameters $\varepsilon_{1}$ and $\varepsilon_{2}$ on the critical range for chaotic vibration in the checkerboard buckling mode system, Figure 5 is presented. The parameters for the system are set as, $\bar{f}=1300$, $\bar{c}=0.01$, $\bar{\omega }=4$.

As shown in Figure 5a, the system exhibits a stable 1-periodic vibration when it lies between 0.01 and 0.036. At this point, since $\varepsilon_{1}$ is small, the potential well depth is relatively low (as shown in Figure 3). As the energy can easily overcome the potential barrier, the structure undergoes inter-well vibration. When $\varepsilon_{1}$ increases to 0.037, the structure undergoes chaotic vibration, and this process terminates when $\varepsilon_{1}$ = 0.059. As $\varepsilon_{1}$ further increases, the system recovers to a stable 1-periodic motion. At this point, since $\varepsilon_{1}$ is large, the potential well depth is high (as shown in Figure 3). Because the energy is insufficient to overcome the potential barrier, the structure undergoes intra-well vibration. Similarly, system response to variations in $\varepsilon_{2}$ are presented in Figure 5b. A comparison between Figure 5a,b reveals that the influences of the pre-strain parameters $\varepsilon_{1}$ and $\varepsilon_{2}$ on the dynamic stability of the system exhibit a clear symmetric characteristic.

By investigating the effect of pre-strain on the system, it can be concluded that as the pre-strain increases, the potential well depth increases, and the system undergoes a transition from period-1 to chaos and then back to period-1. When the pre-strain is small, the structure is prone to inter-well vibration because the energy is sufficient to cross the potential barrier; when chaos occurs, the structure vibrates in the cross-well. When the pre-strain is large, the structure is prone to intra-well vibration due to insufficient energy to cross the potential barrier. The results are consistent with the findings on one-dimensional buckling structures reported by Bi et al. [32]. While quantitative discrepancies exist in numerical simulations due to differences in parameters, qualitative similarities remain evident. For instance, both studies indicate that as pre-strain increases, dynamic behavior evolves from period-1 inter-well oscillations to chaos, eventually returning to period-1 intra-well oscillations. This observation provides a feasible strategy for modulating dynamic behaviors through pre-strain adjustment.

### 3.3. The Effect of the Young’s Moduli of the Substrate

**Figure 6:**
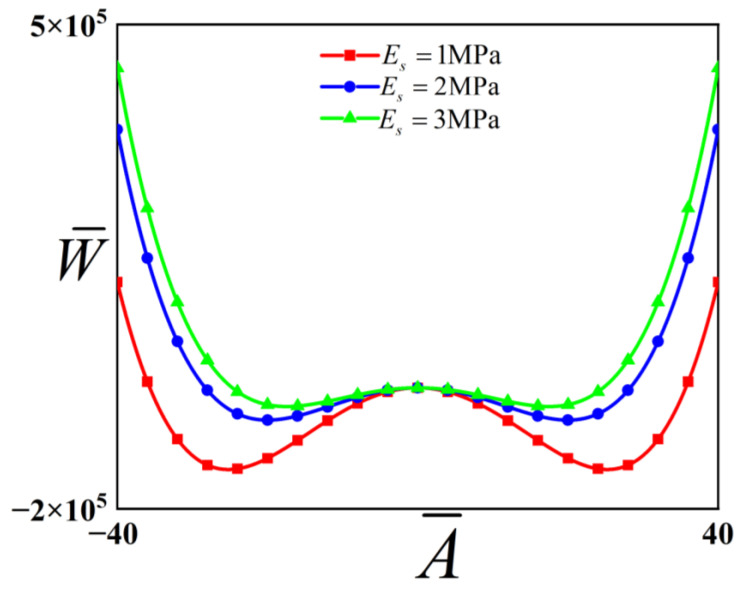
Potential energy functions of the checkerboard buckling mode structure.

To investigate the influence of the Young’s modulus of the substrate on the potential energy function of the system. The influence of the Young’s modulus of substrate parameters on this potential energy function is explored, with the results visualized in Figure 6. The physical parameters of the structure are defined as Song et al. [10]: $E_{f}=130\;\mathrm{GPa}$, $v_{f}=0.27$, $v_{s}=0.48$, $h_{f}\;=10^{-7}\;m$, $\varepsilon_{1}\;=0.05$, $\varepsilon_{2}\;=0.05$.

It can be observed from Figure 6 that with the increase in the Young’s modulus of the substrate, the position of the stable equilibrium point is shifted closer to the initial dimensionless displacement, and the height of the potential barrier is correspondingly reduced. Meanwhile, the distance between the two stable equilibrium points is decreased, and the inter-well distance is also diminished. Consequently, the energy required for the system to perform inter-well vibration is lowered, and it is more likely that inter-well vibration occurs.

**Figure 7:**
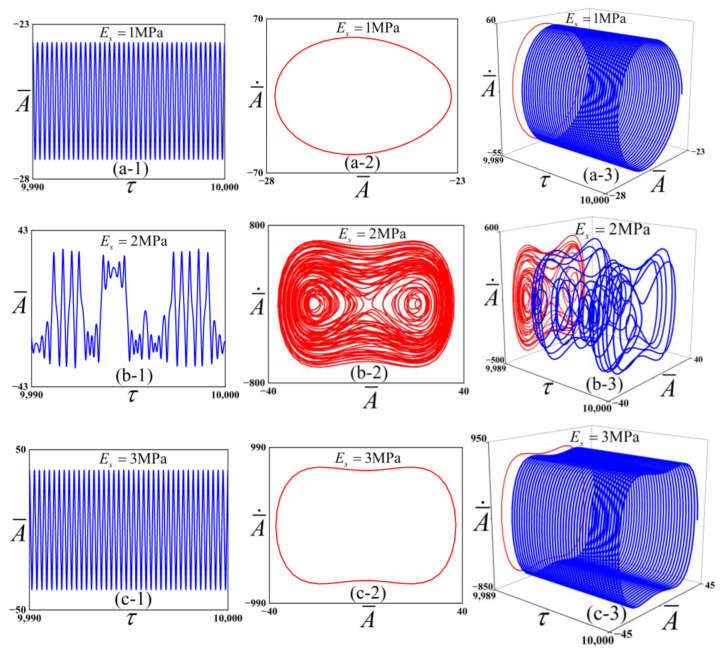
Numerical simulation results for the checkerboard buckling mode structure. Subplots (**a-1**), (**a-2**), and (**a-3**) correspond to the case of $E_{s}=1\;\mathrm{MPa}$, presenting the dimensionless displacement time history, phase portrait, and three-dimensional projection, respectively. Similarly, rows (**b-1**–**b-3**) and (**c-1**–**c-3**) display the corresponding results for $E_{s}=2\;\mathrm{MPa}$ and $E_{s}=3\;\mathrm{MPa}$, respectively.

To preliminarily investigate the influence of different $E_{s}$ values on the system’s dynamic behavior, we performed numerical simulations with the results presented in Figure 7. The parameters of the checkerboard buckling mode system were set as $\bar{f}=1315$, $\bar{c}=0.01$, $\bar{\omega }=4$. The parameter $E_{s}$ takes the values 1 MPa, 2 MPa, 3 MPa.

Figure 7 reveals three distinct dynamic regimes. For $E_{s}=1\;\mathrm{MPa}$, the system exhibits periodic-1 oscillations. Combined with Figure 6, due to the small Young’s modulus of the substrate, the potential well depth is relatively large. The energy of the system is insufficient to overcome the potential barrier, and the structure undergoes intra-well vibration at this point. As $E_{s}$ increases to 2 MPa, the system bifurcates into a chaotic state, characterized by an aperiodic and irregular displacement response. The structure vibrates in the cross-well. Finally, at $E_{s}=3\;\mathrm{MPa}$, the dynamics revert to periodic-1 vibrations. Combined with Figure 6, at this point, since the Young’s modulus of the substrate is relatively large, the potential well depth is relatively small. The system’s energy can easily overcome the potential barrier; thus, the structure undergoes intra-well vibration.

**Figure 8:**
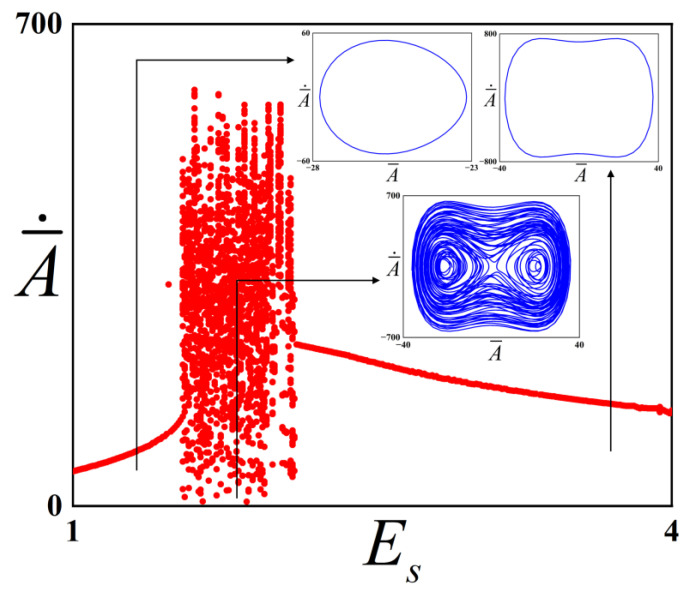
Bifurcation diagram of the checkerboard buckling mode structure with respect to the Young’s modulus of the substrate $E_{s}$. The red dots represent the bifurcation diagrams, while the blue lines represent the phase diagrams under different parameters.

It aims to explore the influence of the Young’s modulus of the substrate $E_{s}$ of the system on the critical value range of chaotic vibration in the checkerboard buckling mode vibration system. Accordingly, Figure 8 was plotted in this study, where the physical parameters of the checkerboard buckling mode system are $\bar{\omega }=4$, $\bar{f}=1315$, $\bar{c}=0.01$.

It can be observed from Figure 8 that a bifurcation diagram corresponding to the Young’s modulus of the substrate $E_{s}$ in the range of 1~4 has been constructed in this study. When $E_{s}$ increases from 1 MPa to 1.56 MPa, the system is maintained in a stable period-1 state and performs inter-well vibration during this process. As $E_{s}$ continues to increase, the system undergoes chaotic vibration, and the chaotic behavior terminates when $E_{s}$ rises to 3.28 MPa. During this process, the system can vibrate in cross-well. With the further increase of $E_{s}$, the system recovers to a period-1 vibration. Meanwhile, the energy is sufficient to overcome the potential barrier, and the structure undergoes inter-well vibration.

By investigating the effect of the Young’s modulus of the substrate on the system, it can be concluded that as the Young’s modulus increases, the potential well depth decreases, and the system undergoes a transition from period-1 to chaos and then back to period-1. When the Young’s modulus of the substrate is small, the structure is prone to intra-well vibration because the energy is insufficient to overcome the potential barrier; when chaos occurs, the structure vibrates in the cross-well. When the pre-strain is large, the structure is prone to inter-well vibration due to sufficient energy to overcome the potential barrier. The present results are qualitatively consistent with those reported by Bi et al. [32] for one-dimensional buckling structures, though quantitative deviations exist due to parametric differences. Specifically, both studies demonstrate that as the substrate Young’s modulus increases, the dynamic behavior transitions from period-1 intra-well oscillation to chaos and eventually returns to period-1 inter-well oscillation. This agreement suggests a viable strategy for tailoring dynamic response regimes through changing the Young’s modulus of the substrate.

## 4. Conclusions

In this study, the governing equations for the nonlinear vibration of rigid-film structures with a checkerboard wrinkle pattern are established. The fourth-order Runge–Kutta method is employed to solve these governing equations, and the dynamic responses of the system over time under different physical parameters are investigated. Additionally, the variation in the potential energy function under different pre-strains and Young’s modulus of the substrate is analyzed, along with the changes in the critical values and stability of the system’s chaotic vibration under various parameters. Numerical simulations reveal that the excitation frequency and the system’s pre-strain can alter the dynamic behavior of the system.

1. The pre-strain and its influence on the potential energy function exhibit symmetry; as the pre-strain increases, the distance between the two stable equilibrium points of the potential energy function increases, and the inter-well distance and potential barrier height also increase in accordance.
2. As the Young’s modulus of the substrate increases, the distance between the two stable equilibrium points of the potential energy function decreases, and the inter-well distance and potential barrier height also decrease accordingly.
3. When the Young’s modulus of the substrate is small, the energy cannot overcome the potential barrier, and the system undergoes intra-well vibration; when the Young’s modulus of the substrate is large, the energy can overcome the potential barrier, and the system will exhibit inter-well vibration.
4. Under the parameters adopted in this study, the stability of the system is affected by the selection of the Young’s modulus of the substrate and the system pre-strain. For instance, the system may transition from period-1 vibration to chaotic vibration.

This study on the nonlinear dynamics of checkerboard-patterned thin film/substrate structures can effectively prevent flexible electronics from entering unstable dynamic regions due to inappropriate selection of material dimensions and parameters during operation, thereby ensuring reliability and robustness in their performance. This study aims to provide theoretical support for the design of two-dimensional checkerboard rigid film structures.

## Data Availability

The original contributions presented in this study are included in the article. Further inquiries can be directed to the corresponding authors.

## Appendix A

**Table A1:** Explanation of symbols in the text.

Nomenclature	
$h_{f}$	The thickness of the film
$v_{f}$	The Poisson’s ratios of the film
$v_{s}$	The Poisson’s ratios of the substrate
${\bar{E}}_{f}$	The equivalent Young’s modulus of the film
${\bar{E}}_{s}$	The equivalent Young’s modulus of the substrate
w	The out-of-plane displacement function
$x_{1}$	The principal strain directions of the biaxial load
$x_{2}$	The principal strain directions of the biaxial load
t	Time
$A\left(t\right)$	The time-varying wrinkle amplitude of this structure
$k_{1}$	The characteristic wave number along the $x_{1}$ directions
$k_{2}$	The characteristic wave number along the $x_{2}$ directions
$k_{st}$	The wave number at steady state
$W_{b}$	The bending energy density of the film
$U_{b}$	The bending energy of the film
$T_{i}$	The shear stress in different directions
$N_{ij}$	The membrane forces to the membrane strains
$\delta_{ij}$	Kronecker Delta
$\varepsilon_{ij}$	The strain tensor of the film
$\varepsilon_{ij}^{pre}$	The pre-strain of the thin film in each direction
$u_{i}^{f}$	The in-plane displacement of the film
$W_{m}$	The membrane energy density of the film
$U_{m}$	The membrane energy of the film
A	The abbreviation of $A\left(t\right)$
$W_{s}$	The strain energy density of the substrate
$\sigma_{ij}$	The stress of the substrate
$U_{s}$	The strain energy of the substrate
$U_{total}$	The total energy
$\dot{A}\left(t\right)$	The first derivative of $A\left(t\right)$ t
$\ddot{A}\left(t\right)$	The second derivative of $A\left(t\right)$ t
E	The total kinetic energy of the film/substrate structure
$\rho_{f}$	The density of the film
$U_{work}$	The work performed by external forces
c	The damping term
$f_{m}\left(t\right)$	The external excitation
f	The amplitude of the external excitation
ω	The frequency of the external excitation
$\bar{\omega }$	The dimensionless frequency of external excitation
τ	The dimensionless time
$\bar{c}$	The dimensionless damping coefficient
$\bar{W}$	The potential energy function of the dimensionless System
